# Allogeneic hematopoietic stem cell transplantation after mogamulizumab in T-cell lymphoma patients: a retrospective analysis

**DOI:** 10.1007/s12185-024-03753-9

**Published:** 2024-03-27

**Authors:** Mary Jo Lechowicz, Christy Smith, Robert Ristuccia, Karen Dwyer

**Affiliations:** 1 The Winship Cancer Institute, Emory University, Atlanta, GA USA; 2 Medical Affairs Oncology, Kyowa Kirin, Inc, Princeton, NJ USA; 3 Medical Affairs Oncology, Kyowa Kirin, Inc, Princeton, NJ USA; 4 Medical Sciences, Kyowa Kirin, Inc, Princeton, NJ USA

**Keywords:** Mogamulizumab, T cell, Lymphoma, Transplant

## Abstract

Allogeneic hematopoietic stem cell transplantation (allo-HSCT) is an important therapy for patients with T-cell lymphomas, including cutaneous T-cell lymphoma (CTCL), adult T-cell lymphoma (ATL), and peripheral T-cell lymphoma (PTCL). Mogamulizumab is an anti-CCR4 antibody that has been associated with an increased risk of transplant-related complications in retrospective analyses of ATL, particularly when administered within 50 days before transplantation. This post hoc analysis of 3 clinical trials examined safety and outcome data for 32 patients with CTCL (*n* = 23), ATL (*n* = 7), or PTCL (*n* = 2) who underwent allo-HSCT after mogamulizumab treatment. Overall, 22 patients (69%) were known to have graft-versus-host disease (GVHD), 8 patients (25%) did not report GVHD, and 2 patients (6%) had unknown GVHD status. Fourteen patients with known GVHD underwent transplantation between 50 and 365 days after their last dose of mogamulizumab, while 2 underwent transplantation within 50 days after treatment. Based on this limited evidence, GVHD was not associated with the time interval from last mogamulizumab dose to transplantation.

**Supplementary Information:**

The online version contains supplementary material available at 10.1007/s12185-024-03753-9.

## Introduction

Non-Hodgkin lymphomas (NHLs) have a global incidence of approximately 3% [1]. T-cell lymphomas account for approximately 10% to 15% of NHLs and encompass a variety of heterogenous disease subtypes [2]. Peripheral T-cell lymphoma (PTCL) originates from mature T cells and makes up approximately 6% of T-cell lymphoma cases [2, 3]. Adult T-cell leukemia/lymphoma (ATL) is a rare and aggressive subtype of PTCL associated with infection by human T-cell lymphotropic virus type 1, which results in cutaneous lesions in approximately half of cases [3]. Cutaneous T-cell lymphoma (CTCL) only accounts for approximately 4% of T-cell lymphoma cases and can affect skin, blood, lymph nodes, or internal organs [2, 3]. Subtypes of CTCL include mycosis fungoides (MF), which is characterized by skin patches, plaques, or tumors, and Sézary syndrome (SS), which is a rare and aggressive subtype characterized by erythroderma, lymphadenopathy, and blood involvement [3].

Allogeneic hematopoietic stem cell transplantation (allo-HSCT) is an important therapeutic option for patients with relapsed or refractory PTCL and ATL [4]. While allo-HSCT has led to prolonged remission in patients with CTCL, posttransplant patients can exhibit increased rates of relapse and graft-versus-host disease (GVHD) [4, 5]. Therefore, in CTCL, allo-HSCT is generally limited to young, otherwise healthy patients with aggressive, advanced-stage disease [5].

Mogamulizumab is an anti-CCR4 antibody that demonstrated safety and efficacy in the treatment of adult patients with relapsed or refractory MF or SS following at least one prior systemic therapy in the phase 3 MAVORIC study [6]. It has also been studied in patients with ATL and PTCL [7, 8]. CCR4 is expressed by regulatory T (Treg) cells, which are responsible for the immune response in GVHD, suggesting that mogamulizumab’s anti-CCR4 activity could exacerbate GVHD by depleting Treg cells [9]. Following mogamulizumab approval in Japan, a retrospective analysis found that mogamulizumab-exposed patients with ATL had an increased risk of grade 3–4 acute GVHD [9]. The results also indicated that risk of GVHD-related complications increased and clinical outcomes were negatively impacted in patients who received their last dose of mogamulizumab < 50 days before allo-HSCT compared to patients with longer intervals. Additional retrospective studies have also suggested a role for mogamulizumab in the development of transplant complications following allo-HSCT [10–13]. These studies evaluated the approved Japanese mogamulizumab dose of 1 mg/kg once weekly for 8 weeks, while the international studies retrospectively reviewed here evaluated doses of 1 mg/kg once weekly for 4 weeks, then twice weekly for subsequent cycles [6, 10–16].

The objective of this post hoc analysis was to report safety and outcome data for patients with CTCL, ATL, or PTCL who underwent allo-HSCT after receiving mogamulizumab.

## Materials and methods

This was a post hoc analysis of 3 international clinical trials (NCT01611142, NCT01626664, and NCT01728805) in patients with CTCL, ATL, or PTCL who were randomized to receive mogamulizumab 1 mg/kg IV weekly for 28 days and then biweekly during subsequent cycles [6, 14, 15]. Full methodologies for these clinical trials have been previously published.

This retrospective study evaluated patients who underwent allo-HSCT after receiving mogamulizumab with a transplant cutoff date of September 1, 2017. Patient records were examined to describe baseline characteristics and transplant details, including human leukocyte antigen matching, allo-HSCT conditioning regimens, GVHD prophylaxis regimens, and GVHD characteristics. All analyses presented are descriptive. No hypothesis testing was conducted given the small sample sizes and the retrospective nature of the study. Original studies obtained Institutional Review Board Approval at the time they were conducted.

## Results

### Baseline characteristics

Of 271 patients who received mogamulizumab in the 3 clinical trials, 32 patients underwent allo-HSCT after mogamulizumab treatment between 2013 and 2017 (CTCL, *n* = 23; ATL, *n* = 7; PTCL, *n* = 2), and their baseline characteristics are presented in Table 1. Twenty-two patients were identified in the United States, 3 in the United Kingdom, 2 each in France and Spain, and 1 each in Japan, the Netherlands, and Switzerland.

**Table 1:** Characteristics of patients who underwent Allo-HSCT after mogamulizumab treatment

		**All patients** **(*N* = 32)**	**CTCL**^**a**^ **(*n* = 23)**	**ATL** **(*n* = 7)**	**PTCL** **(*n* = 2)**
**Age, years**	Mean (SD)	53.0 (13.1)	57.3 (10.2)	46.3 (13.8)	31.0 (12.7)
	Median (range)	56.0 (22.0, 76.0)	59.0 (38.0, 76.0)	50.0 (23.0, 63.0)	31.0 (22.0, 40.0)
**Male, n (%)**		18 (60.0)	14 (60.0)	2 (30.0)	2 (100.0)
**Number of moga infusions**	Mean (SD)	13.0 (13.0)	13.3 (13.3)	8.3 (4.0)	26.0 (26.9)
	Median (range)	8.0 (2.0, 49.0)	8.0 (2.0, 49.0)	8.0 (4.0, 15.0)	26.0 (7.0, 45.0)
**Time from last dose to transplant, days**	Mean (SD)	306.8 (197.7)	301.8 (180.0)	313.1 (229.1)	343.0 (422.8)
	Median (range)	273.5 (16.0, 805.0)	282.0 (16.0, 677.0)	192.0 (169.0, 805.0)	343.0 (44.0 642.0)

*ATL* adult T-cell leukemia/lymphoma, *CTCL* cutaneous T-cell lymphoma, *HSCT* hematopoietic stem cell transplantation, *MF* mycosis fungoides, *moga* mogamulizumab, *PTCL* peripheral T-cell lymphoma, *SD* standard deviation

^a^Includes 1 patient with transformed MF

Among the 23 patients with CTCL, 13 (57%) had a diagnosis of MF, and 10 (43%) had a diagnosis of SS. Thirteen patients (57%) with CTCL were stage IVA at study entry, 1 of whom had transformed MF with primary stage IV disease. The mean (SD) number of mogamulizumab infusions was 11.3 (10.8) in patients with MF (median [range] 9 [2, 43]) and 15.8 (16.2) in patients with SS (median [range] 8 [3, 49]). Among patients with ATL, 3 patients (43%) each had lymphomatous or acute disease, and 1 patient had chronic disease. Four patients (57%) with ATL were stage IV at study entry, 1 patient was stage II, and 2 patients did not have reported stages. Two patients with PTCL were included in this study, one of whom had stage III disease at study entry and the other of whom had stage IV disease.

### Allogeneic HSCT

**Fig. 1:**
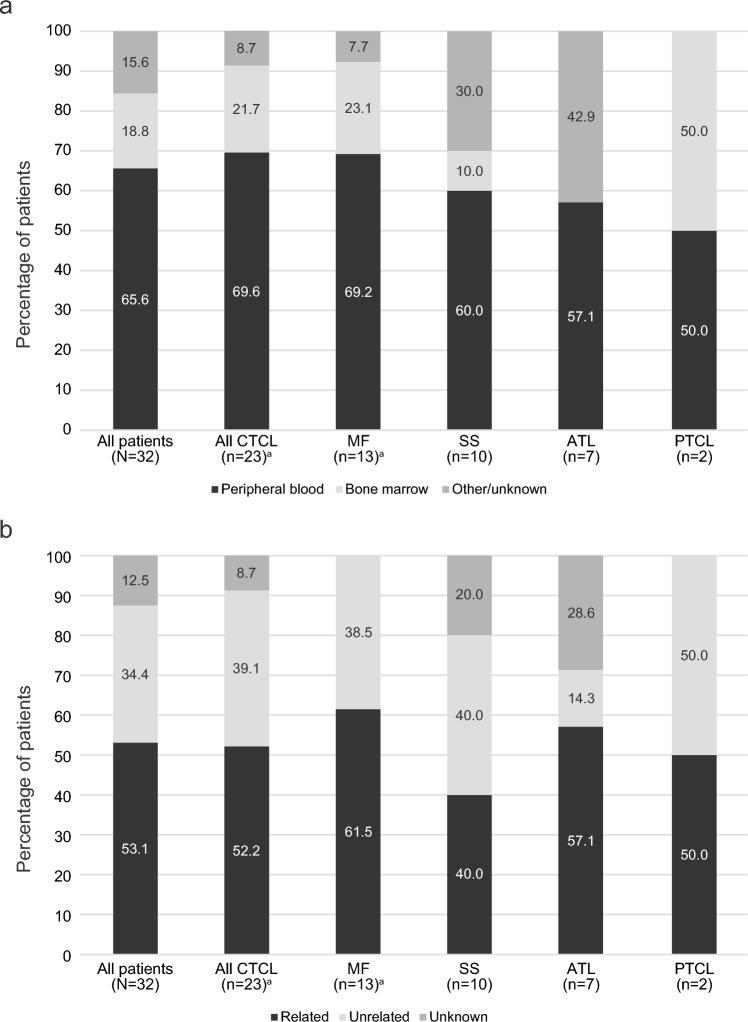
**a** Allo-HSCT donor transplant source and (**b**) donor type. *ATL* adult T-cell leukemia/lymphoma, *CTCL* cutaneous T-cell lymphoma, *HSCT* hematopoietic stem cell transplantation, *MF* mycosis fungoides, *PTCL* peripheral T-cell lymphoma, *SS* Sézary syndrome. ^a^Includes one patient with transformed MF

**Fig. 2:**
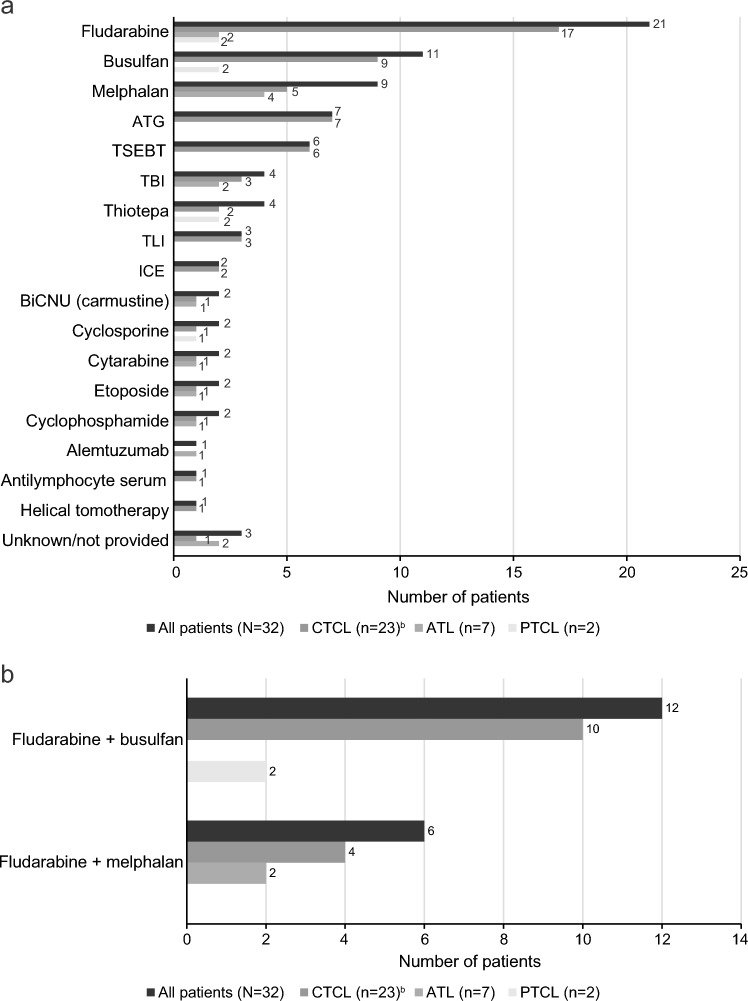
**a** Agents used in conditioning regimens^a^. **b** Selected conditioning regimen combinations. *ATG* antithymocyte globulin, *ATL* adult T-cell lymphoma, *CTCL* cutaneous T-cell lymphoma, *ICE* ifosfamide, carboplatin, etoposide, *MF* mycosis fungoides, *PTCL* peripheral T-cell lymphoma, *TBI* total body irradiation, *TLI* total lymphoid irradiation, *TSEBT* total skin electron beam therapy. ^a^Reported agents based on retrospective chart review. Some are known not to be utilized for conditioning (eg, ICE, cyclosporine); some agents may be used in combination. ^b^Includes one patient with transformed MF

**Fig. 3:**
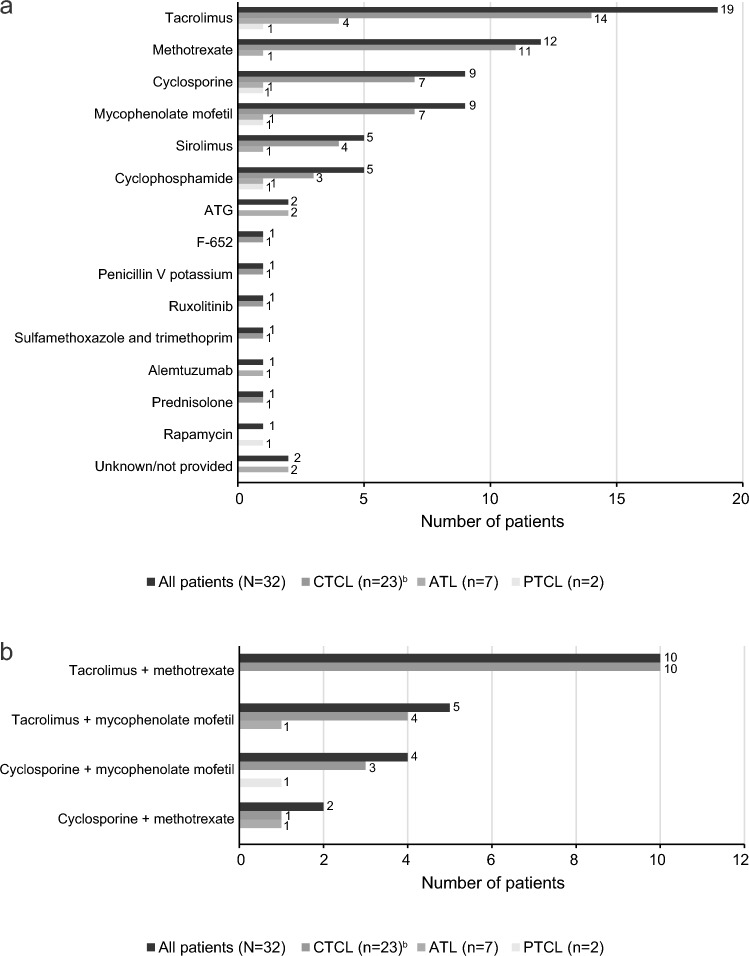
**a** Agents used in the GVHD prophylaxis and/or treatment of GVHD^a^. **b** Selected combination regimens for GVHD prophylaxis and/or Treatment. *ATG* antithymocyte globulin, *ATL* adult T-cell lymphoma, *CTCL* cutaneous T-cell lymphoma, *MF* mycosis fungoides, *PTCL* peripheral T-cell lymphoma. ^a^Reported agents based on retrospective chart review; some are not known to be utilized for prophylaxis (e.g., F 652, penicillin V potassium, prednisolone, ruxolitinib, sulfamethoxazole and trimethoprim, and valacyclovir). ^b^Includes one patient with transformed MF

Human leukocyte antigen (HLA) matching was based on peripheral blood for 21 of the 32 patients (66%), bone marrow for 6 patients (19%), and was unknown for 5 patients (16%) (Fig. 1a). HLA-matched donors were related for 17 patients (53%) and unrelated for 11 patients (34%); relationship status was unknown for 4 patients (13%) (Fig. 1b). The most common conditioning regimens used in this patient population were fludarabine (21 patients [66%]), busulfan (11 patients [34%]), and melphalan (9 patients [28%]) (Fig. 2a). Among the 21 patients treated with fludarabine, 12 (57.1%) were also treated with busulfan and 6 (28.6%) were also treated with melphalan (Fig. 2b). The most common GVHD prophylaxis agents used in this patient population were tacrolimus (19 patients [59%]), methotrexate (12 patients [38%]), and cyclosporine (9 patients [28%]) (Fig. 3a). Ten (52.6%) of the patients treated with tacrolimus were also treated with methotrexate and 5 (26.3%) were also treated with mycophenolate mofetil (Fig. 3b).

### GVHD

Overall, 22 patients (69%) experienced GVHD, 8 patients (25%) reported no GVHD, and GVHD status was unknown for 2 patients (**Supplemental **Fig. 1A). Among those patients who experienced GVHD, 6 patients (27%) had grade 3 GVHD, and 2 patients (9%) had grade 4 (**Supplemental **Fig. 1B). Detailed information on the 8 patients with severe GVHD is provided in **Supplementary Table 1**. Both instances of grade 4 GVHD involved the intestinal tract, and 1 of these instances was reported to be steroid refractory. In those patients with grade 3–4 GVHD, 4 patients (50%) had related donors and 4 patients (50%) had unrelated donors. GVHD was reported as acute/hyperacute in 13 instances and chronic in 5 patients (including some patients with both) and was not described in 9 instances. Acute versus chronic status was known for only 11 instances in CTCL, 4 instances in ATL, and 2 instances in PTCL, with most instances being grade 1 acute GVHD. One instance of grade 3 hyperacute GVHD involving the skin along with grade 2 GVHD of the gut was reported in a patient with stage IV PTCL. This patient had undergone allogeneic transplant 44 days after their last mogamulizumab infusion and had conditioning with thiotepa, fludarabine, and busulfan. Tacrolimus and rapamycin were used for GVHD prophylaxis.

Patients with CTCL experienced 3 instances of grade 3–4 acute GVHD and no instances of grade 3–4 chronic GVHD. When MF and SS were examined separately, there were 8 patients (61.5%) with MF with GVHD of any grade (3 [20.0%] with grade 3–4 GVHD) and 8 patients (80%) with SS with GVHD (2 [20.0%]) with grade 3–4 GVHD). Patients with ATL experienced 1 instance of grade 3–4 chronic GVHD and no instances of grade 3–4 acute GVHD. One patient with PTCL experienced grade 3–4 acute GVHD, and no other grade 3–4 GVHD was reported. Seven patients (31.8%) who experienced GVHD had fludarabine + busulfan for conditioning and tacrolimus + methotrexate for GVHD prophylaxis.

**Fig. 4:**
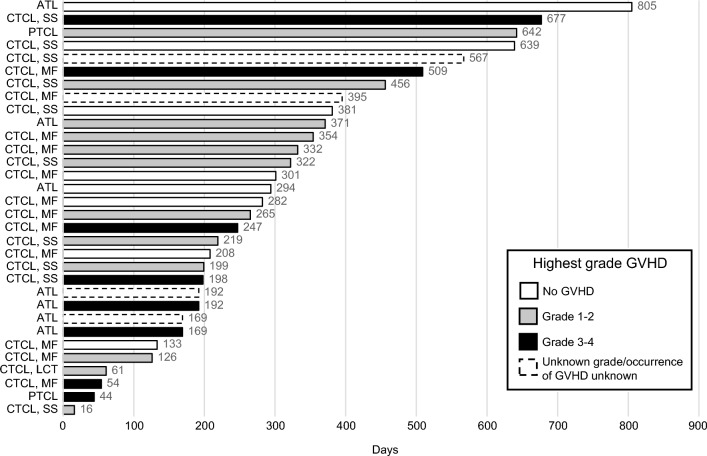
Time since last mogamulizumab infusion

Among all patients with known GVHD, mean (SD) time from last mogamulizumab infusion to transplant was 281.3 (191.7) days (median [range], 233.0 [16.0, 677.0] days). In patients with MF who experienced GVHD, the mean time was 269.6 (150.5) days (median [range], 265.0 [54, 509]), compared with 301.7 (224.5) days (219.0 [16.0, 677.0]) for patients with SS. Mean (SD) time since last mogamulizumab infusion in patients with no grade 1–2, or grade 3–4 GVHD, was 380.4 (227.3), 280.3 (173.8), and 261.3 (221.0), respectively (median [range] 297.5 [133, 805], 293.5 [16, 642], and 195 [44, 677], respectively). Two patients with known GVHD underwent transplant < 50 days after their last dose of mogamulizumab. One patient with CTCL underwent transplant 16 days after mogamulizumab and experienced GVHD of grade 2 in the skin and grade 1 in the skin, abdomen, colon, and eye. One patient with PTCL underwent transplant 44 days after mogamulizumab and experienced GVHD of grade 3 in the skin and grade 2 in the gut (described in detail above). Of 19 patients who underwent transplant between 50 and 365 days after their last dose of mogamulizumab, GVHD occurred in 14 of them, 5 of whom had grade 3–4 GVHD. Six patients who underwent transplant over 365 days after their last dose of mogamulizumab also experienced GVHD. Additional information on time since last mogamulizumab infusion is shown in Fig. 4.

### Follow-up status

At last follow-up, 22 patients (69%) were still alive (median [range] follow-up, 998.0 days [131.0, 1592.0]), and 9 patients had died. Deceased patients had a median (range) survival time at last follow-up of 290.0 days (15.0, 761.0).

## Discussion

In this retrospective study, patients who underwent allo-HSCT after receiving mogamulizumab had generally favorable outcomes. GVHD did not seem to be associated with any meaningful difference in time from last mogamulizumab dose from our limited sample. Most patients with known GVHD underwent transplant between 50 and 365 days after their last mogamulizumab dose, and only 2 patients with known GVHD underwent transplant < 50 days after their last dose of mogamulizumab. The purpose of this study was to examine an experience in other tumor types using mogamulizumab and allogeneic transplant. While it is difficult to answer the question of whether there is any difference in risk between different T-cell tumor subtypes or related to the interval between mogamulizumab treatment and allogeneic transplant with this data set, there may be a suggestion that there is no significant difference, anecdotally. However, several factors critical to the interpretation of the current data set need to be considered. First, the number of patients with transplant < 50 days after their last mogamulizumab treatment is relatively small (only 2 in the present study compared with 42 in the paper published by Fuji et al., likely due to the recommendation provided in that paper to wait at least 50 days before transplant). There were also meaningful differences in the median time between the last dose of mogamulizumab and transplant between the two studies (273.5 days vs 45 days). Overall, these findings suggest a need for further evidence to establish an association between GVHD and interval from last mogamulizumab dose to transplant. It is hoped that the Center for International Blood and Marrow Transplant Research (CIBMTR) study, a noninterventional cohort study evaluating nonrelapse mortality and toxicities in patients with CTCL or ATL treated with mogamulizumab pre- or post-allo-HSCT for patients transplanted that started in 2012, will be able to provide more information about posttransplant outcomes in this population. That said, the time since last mogamulizumab infusion was slightly longer in patients without GVHD relative to those with GVHD, which suggests a possible relationship that should be explored further.

In published literature, patients with ATL who underwent allo-HSCT experienced grade 3–4 acute GVHD at rates of approximately 14% to 20% [17]. Rates of chronic GVHD for patients with PTCL who undergo allo-HSCT have been reported to be between 30 and 60%, but additional findings are limited, as PTCL has relatively low survival rates [18]. Data on the role of allo-HSCT in CTCL are also limited, and no prospective trials have been performed to date. Retrospective registry analyses on allo-HSCT in CTCL include the 2010 and 2021 European Society for Blood and Marrow Transplantation (EBMT) studies, the 2014 CIBMTR study, and the 2020 Japanese Society for Hematopoietic Cell Transplantation study [19–23]. These retrospective registry studies of CTCL report rates of acute GVHD at 35% to 87% and chronic GVHD at 32% to 48% [19–23].

This analysis did not report any instances of grade 3–4 acute GVHD in ATL or chronic GVHD in PTCL. Rates of grade 3–4 acute and chronic GVHD for patients with CTCL were low, further limiting comparison. However, rates of grade 2–4 acute GVHD among patients with CTCL in this analysis (17%) were comparable to rates of grade 2–4 acute GVHD in patients who underwent allo-HSCT and did not receive mogamulizumab reported in the literature (16%), although it is important to note that this information was not available for all patients [24].

While this retrospective analysis did not include translational data to shed light on the potential mechanism, it has been hypothesized that Treg suppression following mogamulizumab administration may contribute to GVHD risk [9]. After mogamulizumab treatment, Tregs remain suppressed for 3 to 4 months [10], which may impair the establishment of immune tolerance after transplant. Consequently, a longer interval between the last mogamulizumab infusion and transplant should lessen the risk as it allows more time for Treg recovery.

This analysis showed that multiple different conditioning regimens were used prior to transplant. Similarly, there was no consistent pattern regarding GVHD prophylaxis. A comparison with the paper published by Fuji et al. on GVHD in patients with ATL who underwent transplant after mogamulizumab treatment would be useful but, unfortunately, the specific regimens were not reported and so this comparison is not possible. These findings suggest that there is little consensus in this analysis regarding the optimal conditioning and prophylaxis regimens for this patient population.

This study has several limitations that should be considered when assessing these results. Patients were drawn from multiple other clinical studies that were not designed to assess transplant outcomes; therefore, the population is very heterogenous. For example, there was a considerable amount of variability in conditioning regimens and treatment/prophylaxis regimens for GVHD. Likewise, the way these regimens were recorded in patient charts was not standardized. Taken together, these factors limit the ability to draw strong conclusions about how they might have affected outcomes. As a retrospective study, some data that could be considered important for this analysis were reported incompletely or were missing. Endpoints of interest that were not available or incomplete included age of allo-HSCT donor, data on patient HLA matching, timing between transplant and onset of GVHD, duration of GVHD, measurements of plasma drug concentrations during washout, and posttransplant survival data. Additionally, patient follow-up was limited, as data for these patients were available for only a short time. The observational nature of this analysis prevents the drawing of firm conclusions around optimal conditioning or GVHD prophylaxis regimens, particularly because some agents may not have been appropriately classified. Moreover, allo-HSCT is more commonly reserved for patients with relapsed or refractory PTCL and ATL rather than CTCL, which comprises the largest percentage of patients in this population. To better assess allo-HSCT outcomes according to FDA postmarketing requirements, investigations seeking prospective data with a larger sample size are currently being conducted with the goal of identifying patients who may benefit from earlier allo-HSCT without advanced disease at diagnosis.

In conclusion, in this cohort of patients who underwent allo-HSCT after treatment with mogamulizumab, patients had generally favorable outcomes, although the small number of patients with transplant < 50 days after their last mogamulizumab infusion limits the conclusions that can be drawn around the effect of time since last drug infusion on GVHD risk. Our retrospective examination of time since infusion in all patients suggests relatively little difference in GVHD risk based on last mogamulizumab infusion in this majority non-ATL population. Multiple different conditioning regimens were used, but there was little consensus on the optimal conditioning regimen for CTCL patients undergoing allo-HSCT. An ongoing observational study being conducted by CIBMTR (NCT04014374) will assess posttransplant outcomes in patients with CTCL or ATL who were treated with mogamulizumab and should provide more data around the impact of the timing of transplant after treatment.

### Supplementary Information

Below is the link to the electronic supplementary material.

Supplementary file1 (DOCX 243 KB)

## Data Availability

The data used for the analyses in this manuscript are available from the corresponding author on reasonable request.

## References

[1] Sung H, Ferlay J, Siegel RL, Laversanne M, Soerjomataram I, Jemal A (2021). Global cancer statistics 2020: GLOBOCAN estimates of incidence and mortality worldwide for 36 cancers in 185 countries. CA Cancer J Clin.

[2] Thandra KC, Barsouk A, Saginala K, Padala SA, Barsouk A, Rawla P (2021). Epidemiology of non-Hodgkin’s lymphoma. Med Sci (Basel).

[3] Phan A, Veldman R, Lechowicz MJ (2016). T-cell lymphoma epidemiology: the known and unknown. Curr Hematol Malig Rep.

[4] Schmitz N, Lenz G, Stelljes M (2018). Allogeneic hematopoietic stem cell transplantation for T-cell lymphomas. Blood.

[5] Dumont M, Peffault de Latour R, Ram-Wolff C, Bagot M, de Masson A. Allogeneic hematopoietic stem cell transplantation in cutaneous T-cell lymphomas. Cancers (Basel). 2020;12(10):2856. 10.3390/cancers12102856. 10.3390/cancers12102856 PMC7601655 33023002

[6] Kim YH, Bagot M, Pinter-Brown L, Rook AH, Porcu P, Horwitz SM (2018). Mogamulizumab versus vorinostat in previously treated cutaneous T-cell lymphoma (MAVORIC): an international, open-label, randomised, controlled phase 3 trial. Lancet Oncol.

[7] Zhang T, Sun J, Li J, Zhao Y, Zhang T, Yang R (2021). Safety and efficacy profile of mogamulizumab (Poteligeo) in the treatment of cancers: an update evidence from 14 studies. BMC Cancer.

[8] POTELIGEO. Prescribing information. Kyowa Kirin, Inc; 2022. https://www.accessdata.fda.gov/drugsatfda_docs/label/2022/761051s015lbl.pdf. Accessed 28 June 2023.

[9] Fuji S, Inoue Y, Utsunomiya A, Moriuchi Y, Uchimaru K, Choi I (2016). Pretransplantation anti-CCR4 antibody mogamulizumab against adult T-cell leukemia/lymphoma is associated with significantly increased risks of severe and corticosteroid-refractory graft-versus-host disease, nonrelapse mortality, and overall mortality. J Clin Oncol.

[10] Kamada Y, Arima N, Hayashida M, Nakamura D, Yoshimitsu M, Ishitsuka K. Prediction of the risk for graft versus host disease after allogeneic hematopoietic stem cell transplantation in patients treated with mogamulizumab. Leuk Lymphoma. 2022:1701–7. 10.1080/10428194.2022.2043300. 10.1080/10428194.2022.2043300 35225126

[11] Iyama S, Sato T, Ohnishi H, Kanisawa Y, Ohta S, Kondo T (2017). A multicenter retrospective study of mogamulizumab efficacy in adult T-cell leukemia/lymphoma. Clin Lymphoma Myeloma Leuk.

[12] Inoue Y, Fuji S, Tanosaki R, Fukuda T (2016). Pretransplant mogamulizumab against ATLL might increase the risk of acute GVHD and non-relapse mortality. Bone Marrow Transplant.

[13] Sugio T, Kato K, Aoki T, Ohta T, Saito N, Yoshida S (2016). Mogamulizumab treatment prior to allogeneic hematopoietic stem cell transplantation induces severe acute graft-versus-host disease. Biol Blood Marrow Transplant.

[14] Zinzani PL, Karlin L, Radford J, Caballero D, Fields P, Chamuleau MED (2016). European phase II study of mogamulizumab, an anti-CCR4 monoclonal antibody, in relapsed/refractory peripheral T-cell lymphoma. Haematologica.

[15] Phillips AA, Fields PA, Hermine O, Ramos JC, Beltran BE, Pereira J (2019). Mogamulizumab versus investigator choice of chemotherapy regimen in relapsed/refractory adult T-cell leukemia/lymphoma. Haematologica.

[16] Ishida T, Joh T, Uike N, Yamamoto K, Utsunomiya A, Yoshida S (2012). Defucosylated anti-CCR4 monoclonal antibody (KW-0761) for relapsed adult T-cell leukemia-lymphoma: a multicenter phase II study. J Clin Oncol.

[17] Ishida T, Hishizawa M, Kato K, Tanosaki R, Fukuda T, Takatsuka Y (2013). Impact of graft-versus-host disease on allogeneic hematopoietic cell transplantation for adult T cell leukemia-lymphoma focusing on preconditioning regimens: nationwide retrospective study. Biol Blood Marrow Transplant.

[18] Huang W-R, Liu D-H (2018). Peripheral T-cell lymphomas: updates in allogeneic hematopoietic stem cell transplantation. Chin Med J (Engl).

[19] Angelov D, Dillon J, Mellerick L, Pender E, Bacon L, Lee G (2022). Allogeneic transplantation in cutaneous T-cell lymphoma: improved outcomes associated with early transplantation and acute graft versus host disease. Bone Marrow Transplant.

[20] Duarte RF, Canals C, Onida F, Gabriel IH, Arranz R, Arcese W (2010). Allogeneic hematopoietic cell transplantation for patients with mycosis fungoides and Sézary syndrome: a retrospective analysis of the Lymphoma Working Party of the European Group for Blood and Marrow Transplantation. J Clin Oncol.

[21] Domingo-Domenech E, Duarte RF, Boumedil A, Onida F, Gabriel I, Finel H, et al. Allogeneic hematopoietic stem cell transplantation for advanced mycosis fungoides and Sézary syndrome. An updated experience of the Lymphoma Working Party of the European Society for Blood and Marrow Transplantation. Bone Marrow Transplant. 2021;56(6):1391–401. 10.1038/s41409-020-01197-3. 10.1038/s41409-020-01197-3 33420392

[22] Lechowicz MJ, Lazarus HM, Carreras J, Laport GG, Cutler CS, Wiernik PH (2014). Allogeneic hematopoietic cell transplantation for mycosis fungoides and Sézary syndrome. Bone Marrow Transplant.

[23] Mori T, Shiratori S, Suzumiya J, Kurokawa M, Shindo M, Naoyuki U (2020). Outcome of allogeneic hematopoietic stem cell transplantation for mycosis fungoides and Sézary syndrome. Hematol Oncol.

[24] Weng W-K, Arai S, Rezvani A, Johnston L, Lowsky R, Miklos D (2020). Nonmyeloablative allogeneic transplantation achieves clinical and molecular remission in cutaneous T-cell lymphoma. Blood Adv.

