# Supplementary material for: Allogeneic hematopoietic stem cell transplantation after mogamulizumab in T-cell lymphoma patients: a retrospective analysis
Source: Int J Hematol. 2024 Mar 27;119(6):736–44. doi: 10.1007/s12185-024-03753-9 (PMC11136860; doi:10.1007/s12185-024-03753-9)
Supplement: Supplementary file 1 — Supplementary file1 (DOCX 243 KB) [file 12185_2024_3753_MOESM1_ESM.docx]

**Supplemental Figure 1. A.** Rates of Known GVHD and **B.** Highest Grade of Known GVHD


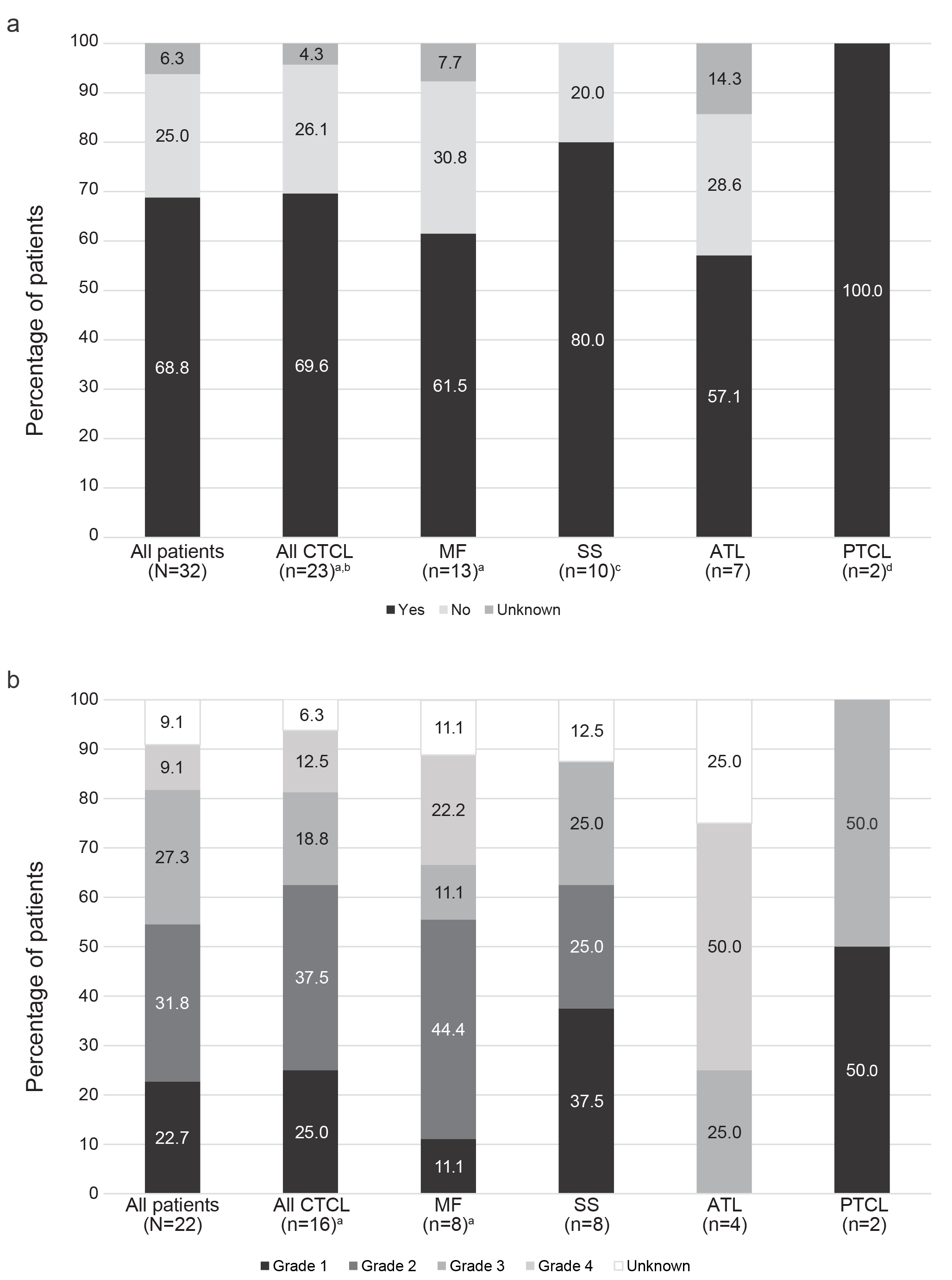


ATL, adult T-cell leukemia/lymphoma; CTCL, cutaneous T-cell lymphoma; GVHD, graft-versus-host disease; MF, mycosis fungoides; PTCL, peripheral T-cell lymphoma.

^a^Includes one patient with transformed MF.

^b^Includes 8 occurrences of acute and 3 occurrences of chronic GVHD.

^c^Includes 2 occurrences of acute/hyperacute and 2 occurrences of chronic GVHD.

# ^d^Includes 2 occurrences of acute and 0 occurrences of chronic GVHD.

Supplementary Table 1. Patients With Severe GVHD

Clinical information on the eight patients with severe GVHD after transplant. All information was captured **as reported** in the patients’ charts.

| **Disease type** | **Age, years** | **Sex** | **Time from last dose of mogamulizumab to transplant, days** | **Conditioning therapy^a^** | **Donor type** | **GVHD prophylaxis therapy** | **GVHD type** |
| --- | --- | --- | --- | --- | --- | --- | --- |
| PTCL | 22 | M | 44 | - Thiotepa - Fludarabine - Busulfan | Allogeneic (unrelated), HLA-matched | - Tacrolimus - Rapamycin | - Grade 3 (skin) - hyperacute - Grade 2 (gut) |
| ATL | 50 | F | 169 | - Fludarabine - Melphalan | Allogeneic (related), HLA-matched | - ATG - Tacrolimus | - Grade 3 (skin, intraoral and intestinal tract) - chronic |
| ATL | 44 | F | 192 | - Melphalan - TBI | Allogeneic (unrelated); HLA-matched | - Sirolimus - Tacrolimus - ATG | - Grade 1 (intestinal tract) - acute; said to have fluctuated from gr 2 to 3, approx  2 years later developed chronic GVHD of oral cavity and skin (grade not provided) |
| CTCL (SS) | 58 | M | 677 | - ICE - TBI | Allogeneic (related); HLA-matched | - Tacrolimus - Methotrexate - Sirolimus - Mycophenolate mofetil | - Grade 3 (skin) - acute |
| CTCL (SS) | 63 | M | 198 | - TSEBT - Fludarabine - Busulfan | Allogeneic (unrelated); HLA-matched | - Tacrolimus - Methotrexate | - Grade 3 (skin) – acute - Grade Unk (intestinal tract) - acute |
| CTCL (MF) | 46 | F | 509 | - Busulfan - Fludarabine - Thiotepa | Allogeneic (related); HLA-matched | - Cyclophosphamide - Tacrolimus - Mycophenolate mofetil - Sirolimus - Ruxolitinib - F-652 | - Grade 4 (intestinal tract) - acute |
| CTCL (MF) | 38 | M | 247 | - Fludarabine - Cyclophosphamide | Allogeneic (related); HLA-matched | - Cyclosporine - Mycophenolate mofetil | - Grade 2 (skin) - acute - Grade 3 (skin, intraoral, eyes, lung, neuromuscular) - chronic |
| CTCL (MF) | 49 | M | 54 | - Fludarabine - Melphalan - TSEBT | Allogeneic (unrelated), HLA-matched | - Tacrolimus - Methotrexate | - Grade 4 (intestinal tract) |

ATG, antithymocyte globulin; ATL, adult T-cell lymphoma; CTCL, cutaneous T-cell lymphoma; F, female; GVHD, graft-vs-host disease; HLA, human leukocyte antigen; ICE, ifosfamide, carboplatin, etoposide; M, male; MF, mycosis fungoides; PTCL, peripheral T-cell lymphoma; SS, Sézary syndrome; TBI, total body irradiation; TSEBT, total skin electron beam therapy; Unk, unknown.

^a^Reported agents based on retrospective chart review. Some are known not to be utilized for conditioning (eg, ICE, cyclosporine); some agents may be used in combination.
